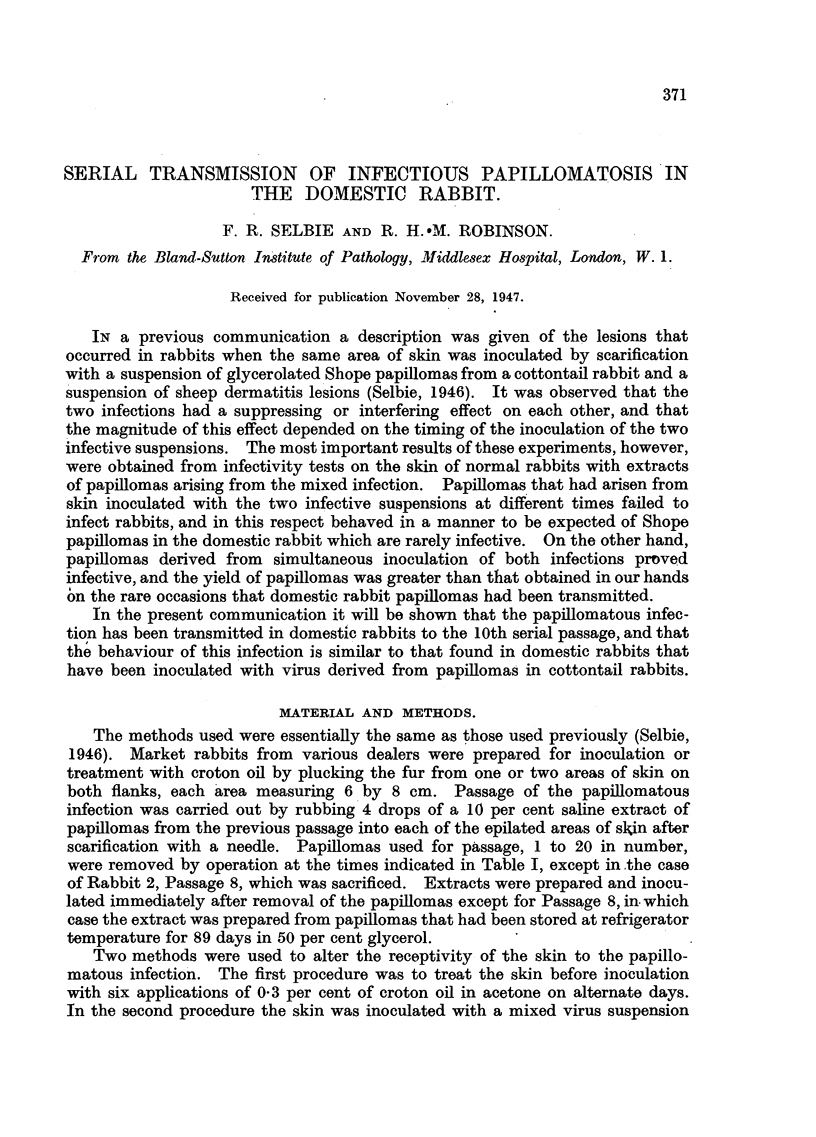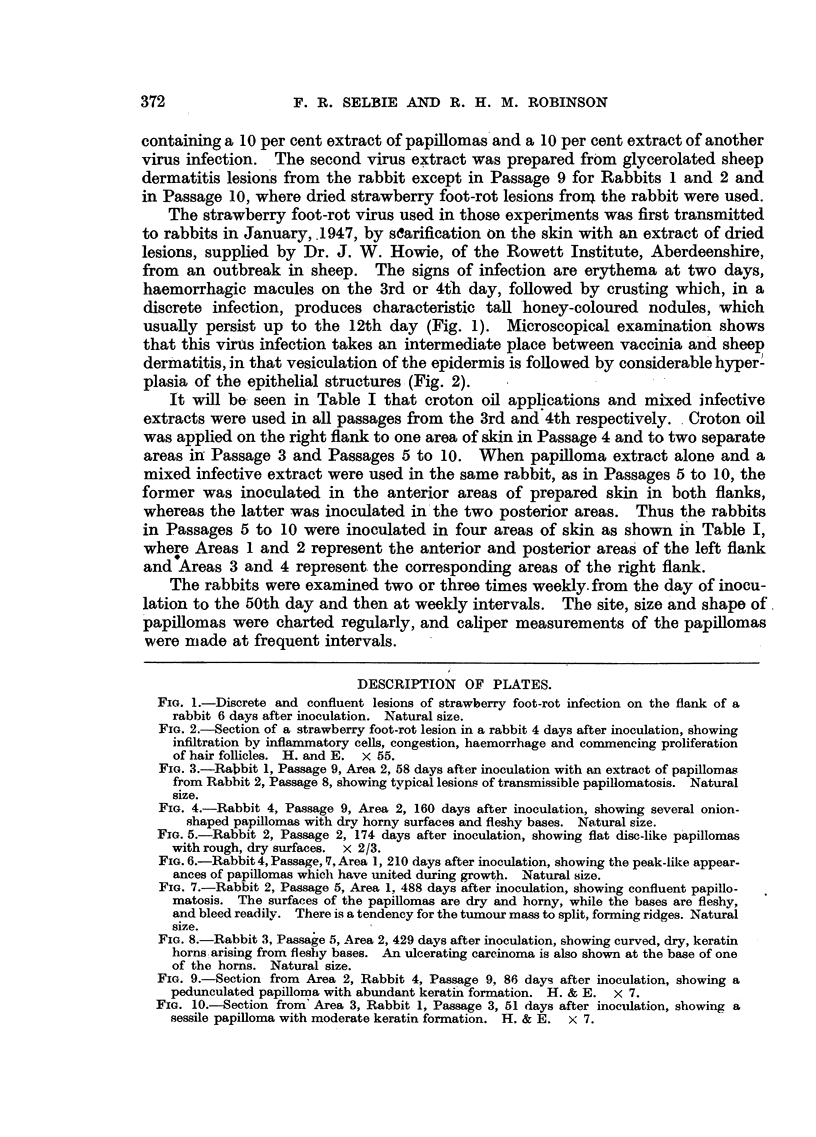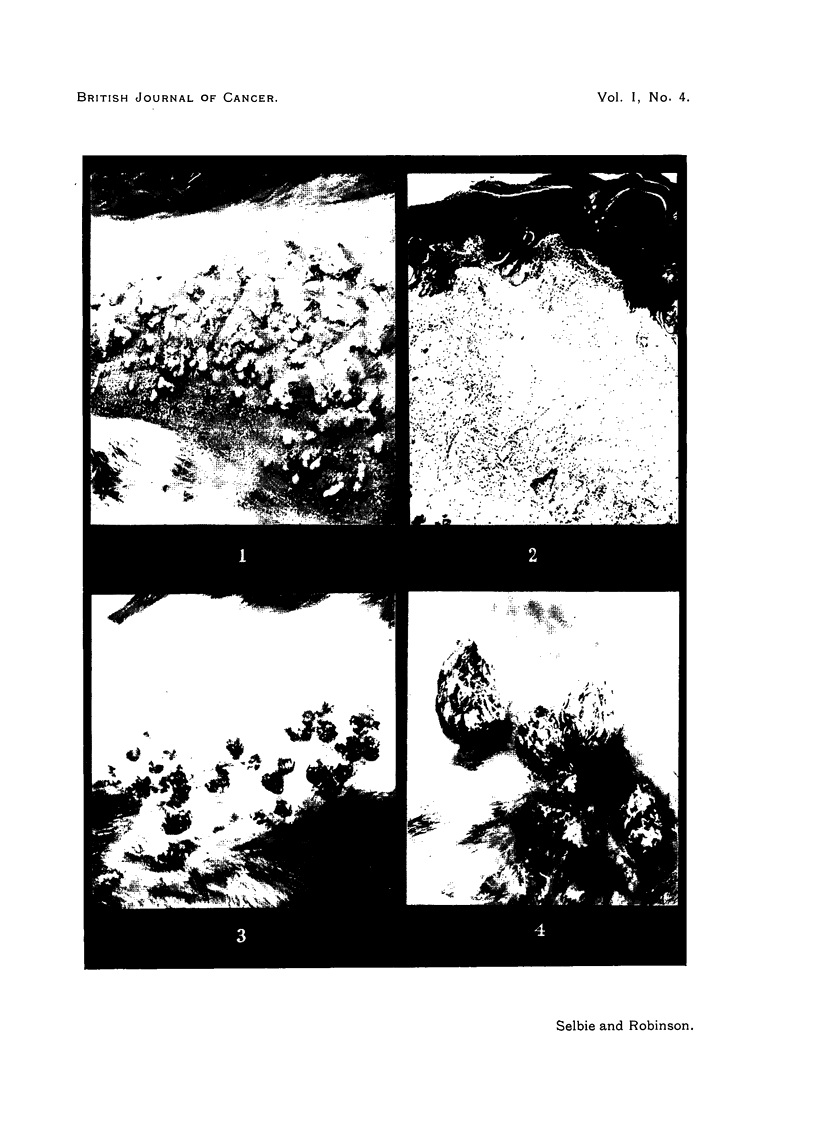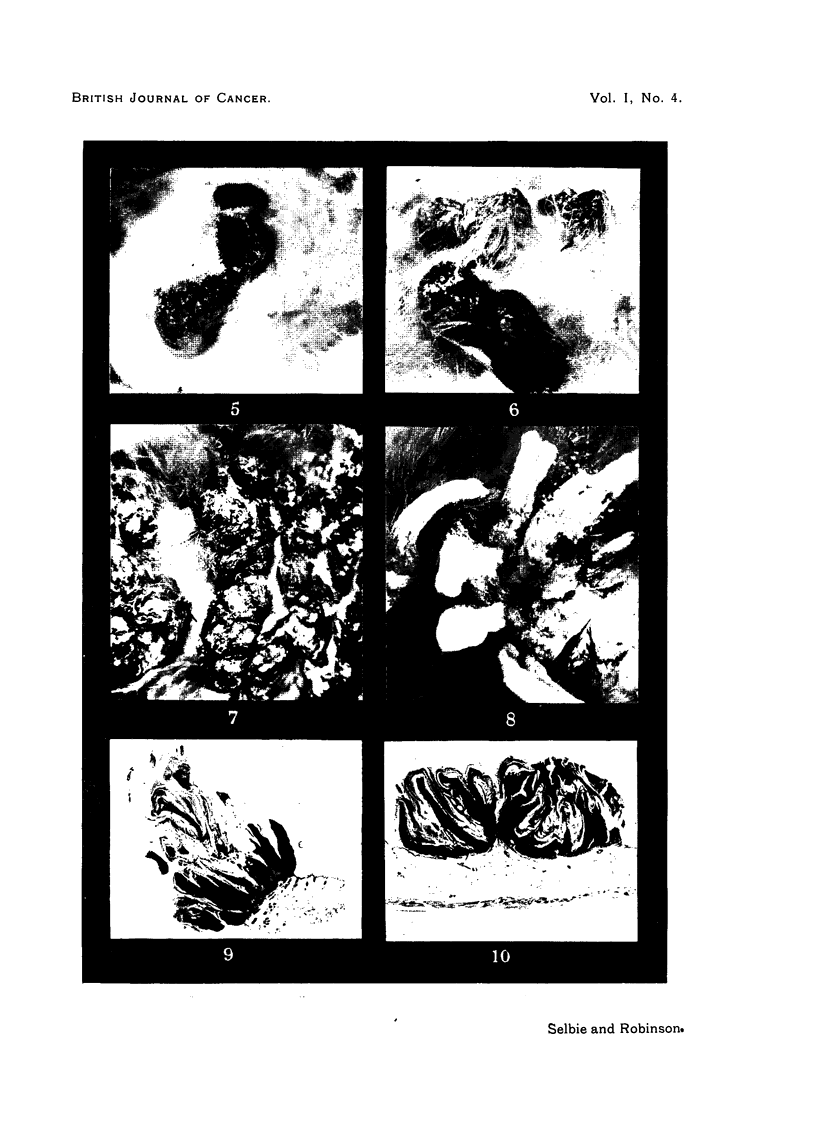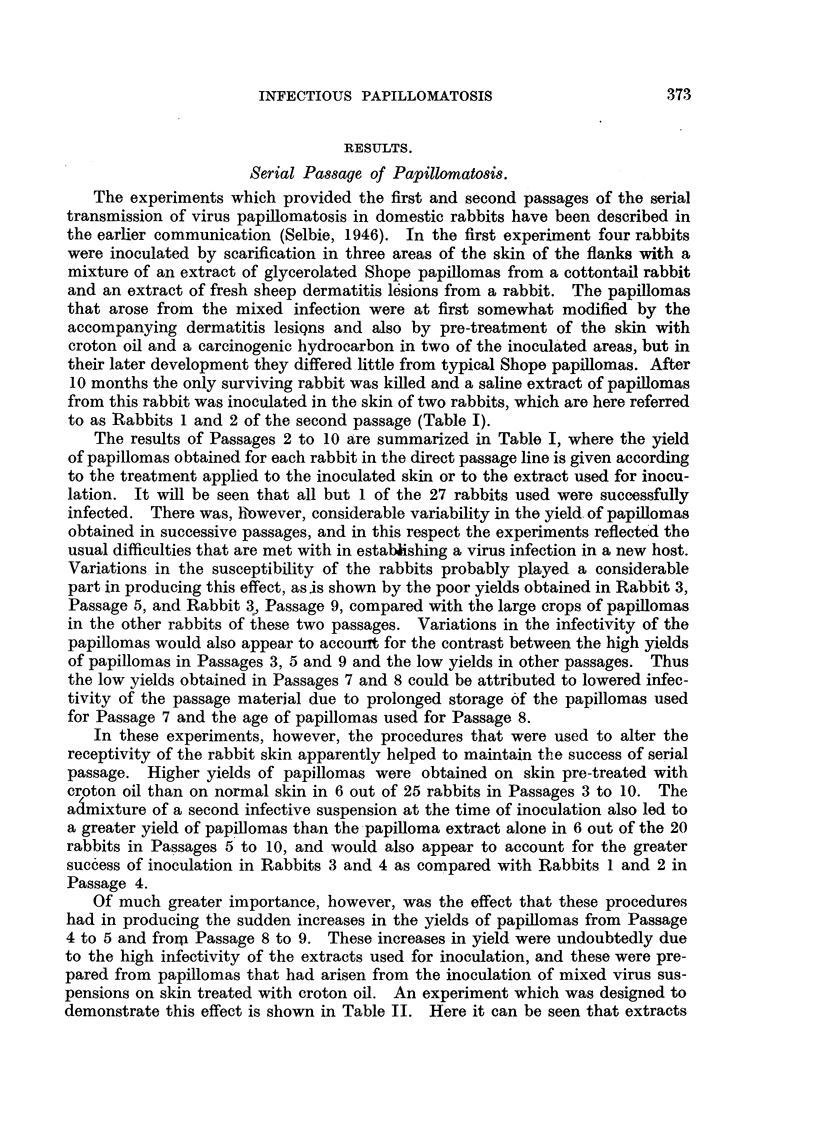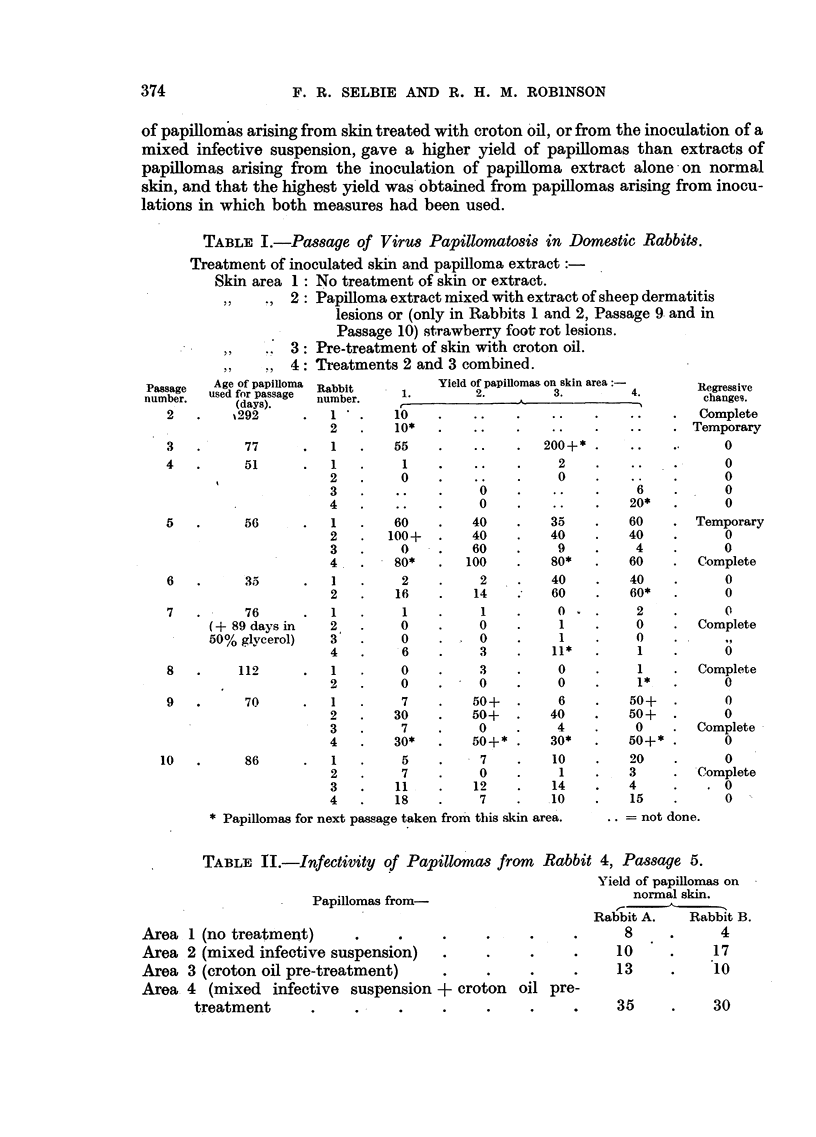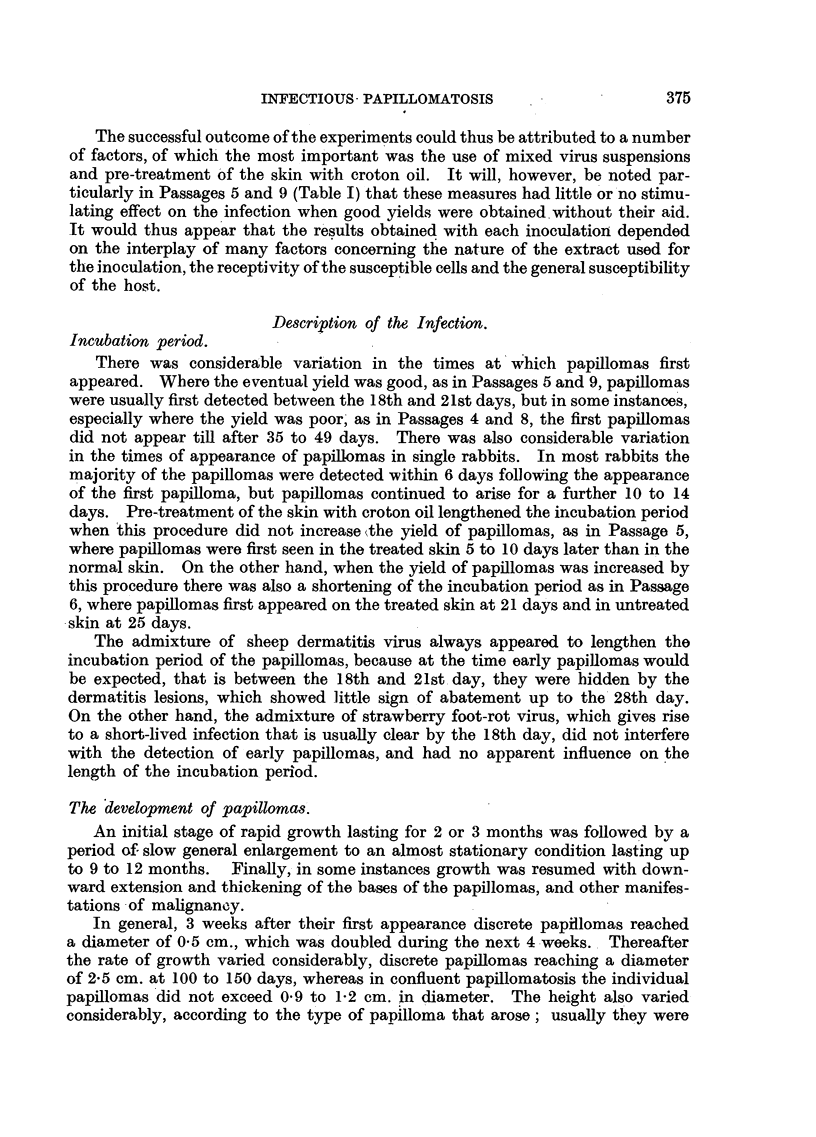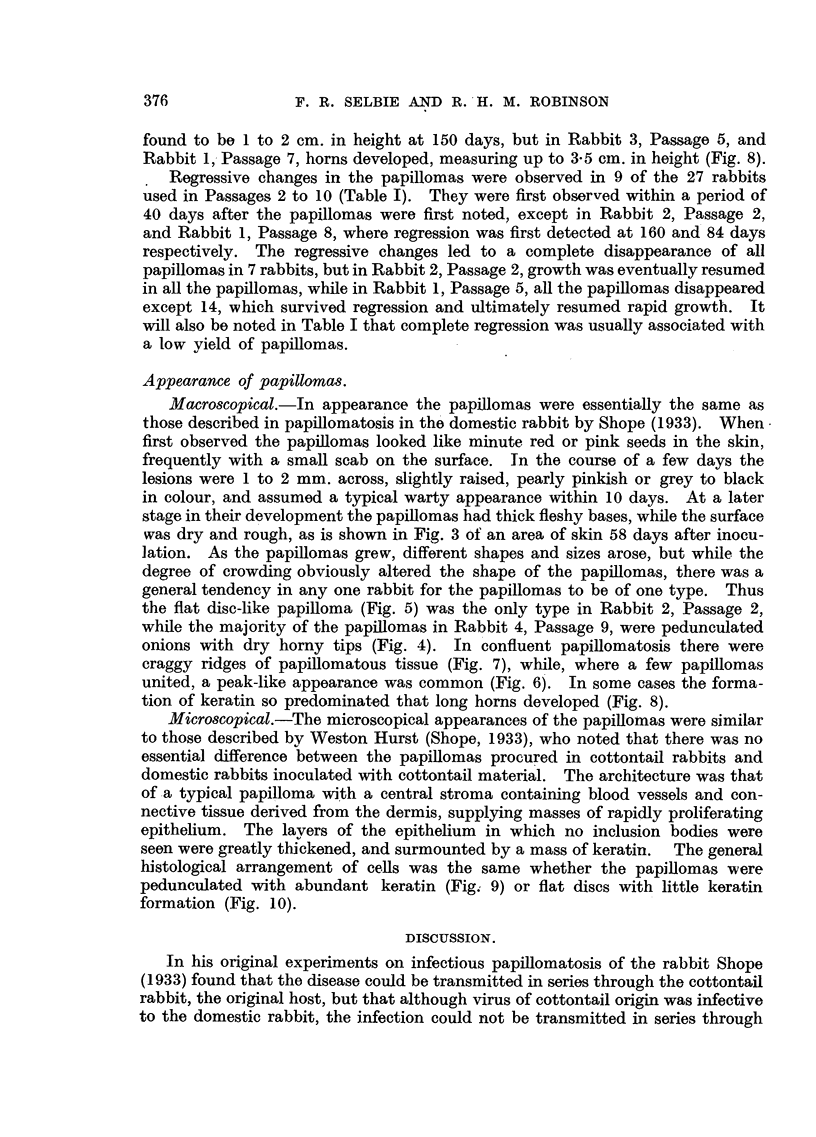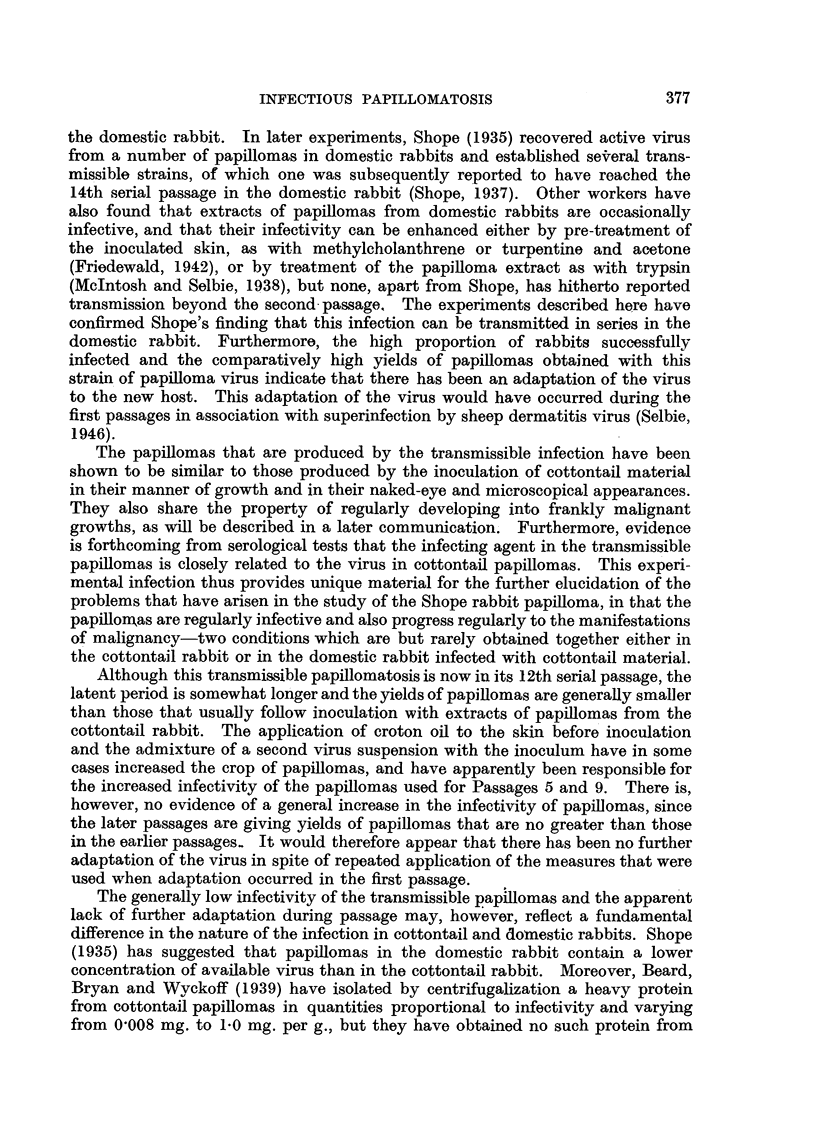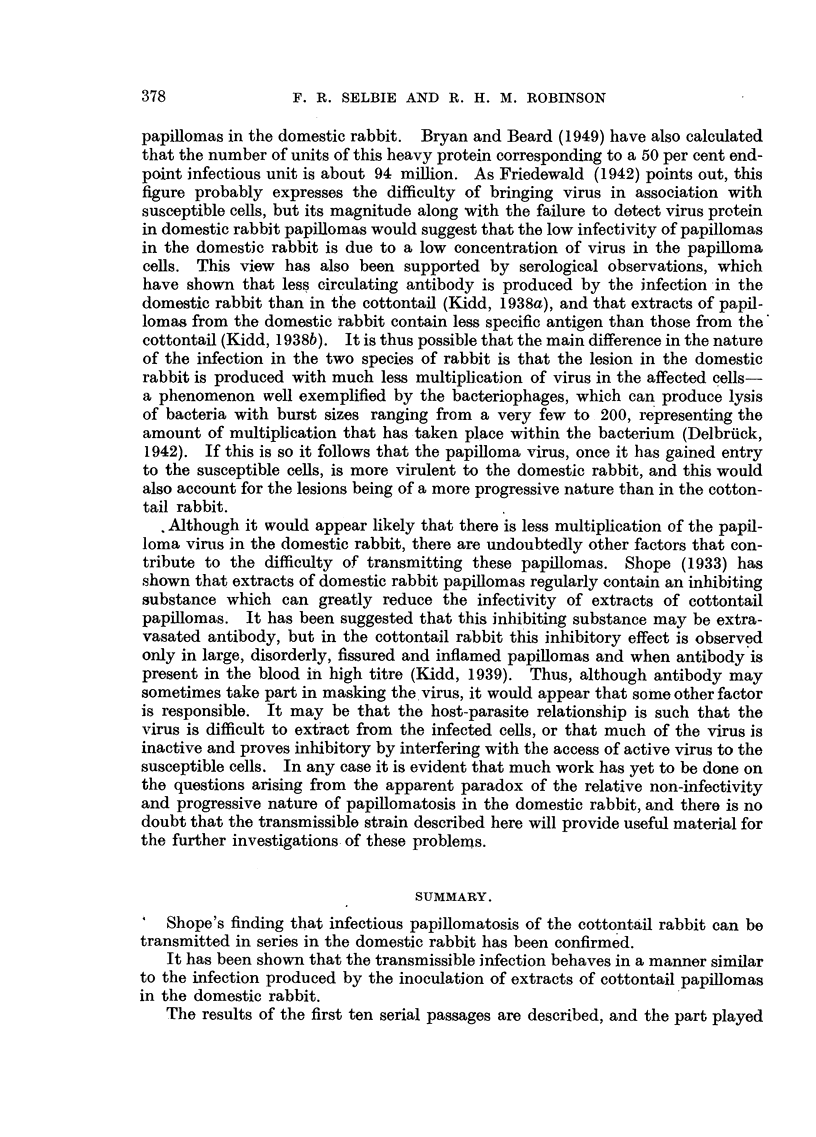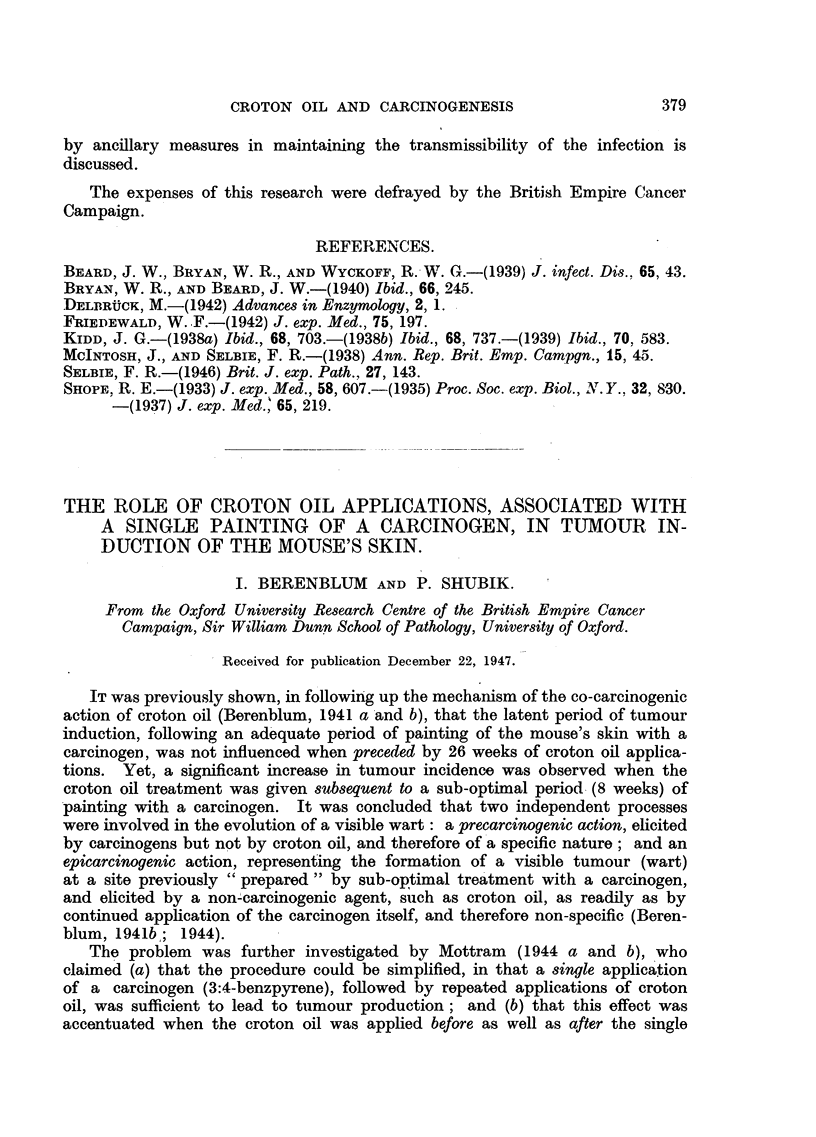# Serial Transmission of Infectious Papillomatosis in the Domestic Rabbit

**DOI:** 10.1038/bjc.1947.34

**Published:** 1947-12

**Authors:** F. R. Selbie, R. H. M. Robinson

## Abstract

**Images:**


### SERIAL TRANSMISSION OF INFECTIOUS PAPILLOMATOSIS IN THE DOMESTIC RABBIT.

F. R. SELBIE AND R. H. M. ROBINSON.

*From the Bland-Sutton Institute of Pathology, Middlesex Hospital, London, W. 1.*

Received for publication November 28, 1947.

In a previous communication a description was given of the lesions that occurred in rabbits when the same area of skin was inoculated by scarification with a suspension of glycerolated Shope papillomas from a cottontail rabbit and a suspension of sheep dermatitis lesions (Selbie, 1946). It was observed that the two infections had a suppressing or interfering effect on each other, and that the magnitude of this effect depended on the timing of the inoculation of the two infective suspensions. The most important results of these experiments, however, were obtained from infectivity tests on the skin of normal rabbits with extracts of papillomas arising from the mixed infection. Papillomas that had arisen from skin inoculated with the two infective suspensions at different times failed to infect rabbits, and in this respect behaved in a manner to be expected of Shope papillomas in the domestic rabbit which are rarely infective. On the other hand, papillomas derived from simultaneous inoculation of both infections proved infective, and the yield of papillomas was greater than that obtained in our hands on the rare occasions that domestic rabbit papillomas had been transmitted.

In the present communication it will be shown that the papillomatous infection has been transmitted in domestic rabbits to the 10th serial passage, and that the behaviour of this infection is similar to that found in domestic rabbits that have been inoculated with virus derived from papillomas in cottontail rabbits.

### MATERIAL AND METHODS.

The methods used were essentially the same as those used previously (Selbie, 1946). Market rabbits from various dealers were prepared for inoculation or treatment with croton oil by plucking the fur from one or two areas of skin on both flanks, each area measuring 6 by 8 cm. Passage of the papillomatous infection was carried out by rubbing 4 drops of a 10 per cent saline extract of papillomas from the previous passage into each of the epilated areas of skin after scarification with a needle. Papillomas used for passage, 1 to 20 in number, were removed by operation at the times indicated in Table I, except in the case of Rabbit 2, Passage 8, which was sacrificed. Extracts were prepared and inoculated immediately after removal of the papillomas except for Passage 8, in which case the extract was prepared from papillomas that had been stored at refrigerator temperature for 89 days in 50 per cent glycerol.

Two methods were used to alter the receptivity of the skin to the papillomatous infection. The first procedure was to treat the skin before inoculation with six applications of 0·3 per cent of croton oil in acetone on alternate days. In the second procedure the skin was inoculated with a mixed virus suspension containing a 10 per cent extract of papillomas and a 10 per cent extract of another virus infection. The second virus extract was prepared from glycerolated sheep dermatitis lesions from the rabbit except in Passage 9 for Rabbits 1 and 2 and in Passage 10, where dried strawberry foot-rot lesions from the rabbit were used.

The strawberry foot-rot virus used in those experiments was first transmitted to rabbits in January, 1947, by scarification on the skin with an extract of dried lesions, supplied by Dr. J. W. Howie, of the Rowett Institute, Aberdeenshire, from an outbreak in sheep. The signs of infection are erythema at two days, haemorrhagic macules on the 3rd or 4th day, followed by crusting which, in a discrete infection, produces characteristic tall honey-coloured nodules, which usually persist up to the 12th day (Fig. 1). Microscopical examination shows that this virus infection takes an intermediate place between vaccinia and sheep dermatitis, in that vesiculation of the epidermis is followed by considerable hyperplasia of the epithelial structures (Fig. 2).

It will be seen in Table I that croton oil applications and mixed infective extracts were used in all passages from the 3rd and 4th respectively. Croton oil was applied on the right flank to one area of skin in Passage 4 and to two separate areas in Passage 3 and Passages 5 to 10. When papilloma extract alone and a mixed infective extract were used in the same rabbit, as in Passages 5 to 10, the former was inoculated in the anterior areas of prepared skin in both flanks, whereas the latter was inoculated in the two posterior areas. Thus the rabbits in Passages 5 to 10 were inoculated in four areas of skin as shown in Table I, where Areas 1 and 2 represent the anterior and posterior areas of the left flank and Areas 3 and 4 represent the corresponding areas of the right flank.

The rabbits were examined two or three times weekly from the day of inoculation to the 50th day and then at weekly intervals. The site, size and shape of papillomas were charted regularly, and caliper measurements of the papillomas were made at frequent intervals.

---

DESCRIPTION OF PLATES.

FIG. 1.—Discrete and confluent lesions of strawberry foot-rot infection on the flank of a rabbit 6 days after inoculation. Natural size.

FIG. 2.—Section of a strawberry foot-rot lesion in a rabbit 4 days after inoculation, showing infiltration by inflammatory cells, congestion, haemorrhage and commencing proliferation of hair follicles. H. and E. × 55.

FIG. 3.—Rabbit 1, Passage 9, Area 2, 58 days after inoculation with an extract of papillomas from Rabbit 2, Passage 8, showing typical lesions of transmissible papillomatosis. Natural size.

FIG. 4.—Rabbit 4, Passage 9, Area 2, 160 days after inoculation, showing several onion-shaped papillomas with dry horny surfaces and fleshy bases. Natural size.

FIG. 5.—Rabbit 2, Passage 2, 174 days after inoculation, showing flat disc-like papillomas with rough, dry surfaces. × 2/3.

FIG. 6.—Rabbit 4, Passage, 7, Area 1, 210 days after inoculation, showing the peak-like appearances of papillomas which have united during growth. Natural size.

FIG. 7.—Rabbit 2, Passage 5, Area 1, 488 days after inoculation, showing confluent papillomatosis. The surfaces of the papillomas are dry and horny, while the bases are fleshy, and bleed readily. There is a tendency for the tumour mass to split, forming ridges. Natural size.

FIG. 8.—Rabbit 3, Passage 5, Area 2, 429 days after inoculation, showing curved, dry, keratin horns arising from fleshy bases. An ulcerating carcinoma is also shown at the base of one of the horns. Natural size.

FIG. 9.—Section from Area 2, Rabbit 4, Passage 9, 86 days after inoculation, showing a pedunculated papilloma with abundant keratin formation. H. & E. × 7.

FIG. 10.—Section from Area 3, Rabbit 1, Passage 3, 51 days after inoculation, showing a sessile papilloma with moderate keratin formation. H. & E. × 7.

1

2

3

4

Selbie and Robinson.

5

6

7

8

9

10

Selbie and Robinson.

### RESULTS.

### *Serial Passage of Papillomatosis.*

The experiments which provided the first and second passages of the serial transmission of virus papillomatosis in domestic rabbits have been described in the earlier communication (Selbie, 1946). In the first experiment four rabbits were inoculated by scarification in three areas of the skin of the flanks with a mixture of an extract of glycerolated Shope papillomas from a cottontail rabbit and an extract of fresh sheep dermatitis lesions from a rabbit. The papillomas that arose from the mixed infection were at first somewhat modified by the accompanying dermatitis lesions and also by pre-treatment of the skin with croton oil and a carcinogenic hydrocarbon in two of the inoculated areas, but in their later development they differed little from typical Shope papillomas. After 10 months the only surviving rabbit was killed and a saline extract of papillomas from this rabbit was inoculated in the skin of two rabbits, which are here referred to as Rabbits 1 and 2 of the second passage (Table I).

The results of Passages 2 to 10 are summarized in Table I, where the yield of papillomas obtained for each rabbit in the direct passage line is given according to the treatment applied to the inoculated skin or to the extract used for inoculation. It will be seen that all but 1 of the 27 rabbits used were successfully infected. There was, however, considerable variability in the yield of papillomas obtained in successive passages, and in this respect the experiments reflected the usual difficulties that are met with in establishing a virus infection in a new host. Variations in the susceptibility of the rabbits probably played a considerable part in producing this effect, as is shown by the poor yields obtained in Rabbit 3, Passage 5, and Rabbit 3, Passage 9, compared with the large crops of papillomas in the other rabbits of these two passages. Variations in the infectivity of the papillomas would also appear to account for the contrast between the high yields of papillomas in Passages 3, 5 and 9 and the low yields in other passages. Thus the low yields obtained in Passages 7 and 8 could be attributed to lowered infectivity of the passage material due to prolonged storage of the papillomas used for Passage 7 and the age of papillomas used for Passage 8.

In these experiments, however, the procedures that were used to alter the receptivity of the rabbit skin apparently helped to maintain the success of serial passage. Higher yields of papillomas were obtained on skin pre-treated with croton oil than on normal skin in 6 out of 25 rabbits in Passages 3 to 10. The admixture of a second infective suspension at the time of inoculation also led to a greater yield of papillomas than the papilloma extract alone in 6 out of the 20 rabbits in Passages 5 to 10, and would also appear to account for the greater success of inoculation in Rabbits 3 and 4 as compared with Rabbits 1 and 2 in Passage 4.

Of much greater importance, however, was the effect that these procedures had in producing the sudden increases in the yields of papillomas from Passage 4 to 5 and from Passage 8 to 9. These increases in yield were undoubtedly due to the high infectivity of the extracts used for inoculation, and these were prepared from papillomas that had arisen from the inoculation of mixed virus suspensions on skin treated with croton oil. An experiment which was designed to demonstrate this effect is shown in Table II. Here it can be seen that extracts of papillomas arising from skin treated with croton oil, or from the inoculation of a mixed infective suspension, gave a higher yield of papillomas than extracts of papillomas arising from the inoculation of papilloma extract alone on normal skin, and that the highest yield was obtained from papillomas arising from inoculations in which both measures had been used.

TABLE I.—*Passage of Virus Papillomatosis in Domestic Rabbits.*

Treatment of inoculated skin and papilloma extract :—
Skin area 1 : No treatment of skin or extract.
,, ,, 2 : Papilloma extract mixed with extract of sheep dermatitis lesions or (only in Rabbits 1 and 2, Passage 9 and in Passage 10) strawberry foot rot lesions.
,, ,, 3 : Pre-treatment of skin with croton oil.
,, ,, 4 : Treatments 2 and 3 combined.

| Passage number. | Age of papilloma used for passage (days). | Rabbit number. | Yield of papillomas on skin area :— 1. | 2. | 3. | 4. | Regressive changes. |
|---|---|---|---|---|---|---|---|
| 2 | 292 | 1 | 10 | .. | .. | .. | Complete |
| | | 2 | 10* | .. | .. | .. | Temporary |
| 3 | 77 | 1 | 55 | .. | 200+* | .. | 0 |
| 4 | 51 | 1 | 1 | .. | 2 | .. | 0 |
| | | 2 | 0 | .. | 0 | .. | 0 |
| | | 3 | .. | 0 | .. | 6 | 0 |
| | | 4 | .. | 0 | .. | 20* | 0 |
| 5 | 56 | 1 | 60 | 40 | 35 | 60 | Temporary |
| | | 2 | 100+ | 40 | 40 | 40 | 0 |
| | | 3 | 0 | 60 | 9 | 4 | 0 |
| | | 4 | 80* | 100 | 80* | 60 | Complete |
| 6 | 35 | 1 | 2 | 2 | 40 | 40 | 0 |
| | | 2 | 16 | 14 | 60 | 60* | 0 |
| 7 | 76 (+ 89 days in 50% glycerol) | 1 | 1 | 1 | 0 | 2 | 0 |
| | | 2 | 0 | 0 | 1 | 0 | Complete |
| | | 3 | 0 | 0 | 1 | 0 | ,, |
| | | 4 | 6 | 3 | 11* | 1 | 0 |
| 8 | 112 | 1 | 0 | 3 | 0 | 1 | Complete |
| | | 2 | 0 | 0 | 0 | 1* | 0 |
| 9 | 70 | 1 | 7 | 50+ | 6 | 50+ | 0 |
| | | 2 | 30 | 50+ | 40 | 50+ | 0 |
| | | 3 | 7 | 0 | 4 | 0 | Complete |
| | | 4 | 30* | 50+* | 30* | 50+* | 0 |
| 10 | 86 | 1 | 5 | 7 | 10 | 20 | 0 |
| | | 2 | 7 | 0 | 1 | 3 | Complete |
| | | 3 | 11 | 12 | 14 | 4 | 0 |
| | | 4 | 18 | 7 | 10 | 15 | 0 |

* Papillomas for next passage taken from this skin area. .. = not done.

TABLE II.—*Infectivity of Papillomas from Rabbit 4, Passage 5.*

| Papillomas from— | Yield of papillomas on normal skin. Rabbit A. | Rabbit B. |
|---|---|---|
| Area 1 (no treatment) | 8 | 4 |
| Area 2 (mixed infective suspension) | 10 | 17 |
| Area 3 (croton oil pre-treatment) | 13 | 10 |
| Area 4 (mixed infective suspension + croton oil pre-treatment | 35 | 30 |

The successful outcome of the experiments could thus be attributed to a number of factors, of which the most important was the use of mixed virus suspensions and pre-treatment of the skin with croton oil. It will, however, be noted particularly in Passages 5 and 9 (Table I) that these measures had little or no stimulating effect on the infection when good yields were obtained without their aid. It would thus appear that the results obtained with each inoculation depended on the interplay of many factors concerning the nature of the extract used for the inoculation, the receptivity of the susceptible cells and the general susceptibility of the host.

### *Description of the Infection.*

*Incubation period.*

There was considerable variation in the times at which papillomas first appeared. Where the eventual yield was good, as in Passages 5 and 9, papillomas were usually first detected between the 18th and 21st days, but in some instances, especially where the yield was poor, as in Passages 4 and 8, the first papillomas did not appear till after 35 to 49 days. There was also considerable variation in the times of appearance of papillomas in single rabbits. In most rabbits the majority of the papillomas were detected within 6 days following the appearance of the first papilloma, but papillomas continued to arise for a further 10 to 14 days. Pre-treatment of the skin with croton oil lengthened the incubation period when this procedure did not increase the yield of papillomas, as in Passage 5, where papillomas were first seen in the treated skin 5 to 10 days later than in the normal skin. On the other hand, when the yield of papillomas was increased by this procedure there was also a shortening of the incubation period as in Passage 6, where papillomas first appeared on the treated skin at 21 days and in untreated skin at 25 days.

The admixture of sheep dermatitis virus always appeared to lengthen the incubation period of the papillomas, because at the time early papillomas would be expected, that is between the 18th and 21st day, they were hidden by the dermatitis lesions, which showed little sign of abatement up to the 28th day. On the other hand, the admixture of strawberry foot-rot virus, which gives rise to a short-lived infection that is usually clear by the 18th day, did not interfere with the detection of early papillomas, and had no apparent influence on the length of the incubation period.

*The development of papillomas.*

An initial stage of rapid growth lasting for 2 or 3 months was followed by a period of slow general enlargement to an almost stationary condition lasting up to 9 to 12 months. Finally, in some instances growth was resumed with downward extension and thickening of the bases of the papillomas, and other manifestations of malignancy.

In general, 3 weeks after their first appearance discrete papillomas reached a diameter of 0·5 cm., which was doubled during the next 4 weeks. Thereafter the rate of growth varied considerably, discrete papillomas reaching a diameter of 2·5 cm. at 100 to 150 days, whereas in confluent papillomatosis the individual papillomas did not exceed 0·9 to 1·2 cm. in diameter. The height also varied considerably, according to the type of papilloma that arose ; usually they were found to be 1 to 2 cm. in height at 150 days, but in Rabbit 3, Passage 5, and Rabbit 1, Passage 7, horns developed, measuring up to 3·5 cm. in height (Fig. 8).

Regressive changes in the papillomas were observed in 9 of the 27 rabbits used in Passages 2 to 10 (Table I). They were first observed within a period of 40 days after the papillomas were first noted, except in Rabbit 2, Passage 2, and Rabbit 1, Passage 8, where regression was first detected at 160 and 84 days respectively. The regressive changes led to a complete disappearance of all papillomas in 7 rabbits, but in Rabbit 2, Passage 2, growth was eventually resumed in all the papillomas, while in Rabbit 1, Passage 5, all the papillomas disappeared except 14, which survived regression and ultimately resumed rapid growth. It will also be noted in Table I that complete regression was usually associated with a low yield of papillomas.

*Appearance of papillomas.*

*Macroscopical.*—In appearance the papillomas were essentially the same as those described in papillomatosis in the domestic rabbit by Shope (1933). When first observed the papillomas looked like minute red or pink seeds in the skin, frequently with a small scab on the surface. In the course of a few days the lesions were 1 to 2 mm. across, slightly raised, pearly pinkish or grey to black in colour, and assumed a typical warty appearance within 10 days. At a later stage in their development the papillomas had thick fleshy bases, while the surface was dry and rough, as is shown in Fig. 3 of an area of skin 58 days after inoculation. As the papillomas grew, different shapes and sizes arose, but while the degree of crowding obviously altered the shape of the papillomas, there was a general tendency in any one rabbit for the papillomas to be of one type. Thus the flat disc-like papilloma (Fig. 5) was the only type in Rabbit 2, Passage 2, while the majority of the papillomas in Rabbit 4, Passage 9, were pedunculated onions with dry horny tips (Fig. 4). In confluent papillomatosis there were craggy ridges of papillomatous tissue (Fig. 7), while, where a few papillomas united, a peak-like appearance was common (Fig. 6). In some cases the formation of keratin so predominated that long horns developed (Fig. 8).

*Microscopical.*—The microscopical appearances of the papillomas were similar to those described by Weston Hurst (Shope, 1933), who noted that there was no essential difference between the papillomas procured in cottontail rabbits and domestic rabbits inoculated with cottontail material. The architecture was that of a typical papilloma with a central stroma containing blood vessels and connective tissue derived from the dermis, supplying masses of rapidly proliferating epithelium. The layers of the epithelium in which no inclusion bodies were seen were greatly thickened, and surmounted by a mass of keratin. The general histological arrangement of cells was the same whether the papillomas were pedunculated with abundant keratin (Fig. 9) or flat discs with little keratin formation (Fig. 10).

### DISCUSSION.

In his original experiments on infectious papillomatosis of the rabbit Shope (1933) found that the disease could be transmitted in series through the cottontail rabbit, the original host, but that although virus of cottontail origin was infective to the domestic rabbit, the infection could not be transmitted in series through the domestic rabbit. In later experiments, Shope (1935) recovered active virus from a number of papillomas in domestic rabbits and established several transmissible strains, of which one was subsequently reported to have reached the 14th serial passage in the domestic rabbit (Shope, 1937). Other workers have also found that extracts of papillomas from domestic rabbits are occasionally infective, and that their infectivity can be enhanced either by pre-treatment of the inoculated skin, as with methylcholanthrene or turpentine and acetone (Friedewald, 1942), or by treatment of the papilloma extract as with trypsin (McIntosh and Selbie, 1938), but none, apart from Shope, has hitherto reported transmission beyond the second passage. The experiments described here have confirmed Shope's finding that this infection can be transmitted in series in the domestic rabbit. Furthermore, the high proportion of rabbits successfully infected and the comparatively high yields of papillomas obtained with this strain of papilloma virus indicate that there has been an adaptation of the virus to the new host. This adaptation of the virus would have occurred during the first passages in association with superinfection by sheep dermatitis virus (Selbie, 1946).

The papillomas that are produced by the transmissible infection have been shown to be similar to those produced by the inoculation of cottontail material in their manner of growth and in their naked-eye and microscopical appearances. They also share the property of regularly developing into frankly malignant growths, as will be described in a later communication. Furthermore, evidence is forthcoming from serological tests that the infecting agent in the transmissible papillomas is closely related to the virus in cottontail papillomas. This experimental infection thus provides unique material for the further elucidation of the problems that have arisen in the study of the Shope rabbit papilloma, in that the papillomas are regularly infective and also progress regularly to the manifestations of malignancy—two conditions which are but rarely obtained together either in the cottontail rabbit or in the domestic rabbit infected with cottontail material.

Although this transmissible papillomatosis is now in its 12th serial passage, the latent period is somewhat longer and the yields of papillomas are generally smaller than those that usually follow inoculation with extracts of papillomas from the cottontail rabbit. The application of croton oil to the skin before inoculation and the admixture of a second virus suspension with the inoculum have in some cases increased the crop of papillomas, and have apparently been responsible for the increased infectivity of the papillomas used for Passages 5 and 9. There is, however, no evidence of a general increase in the infectivity of papillomas, since the later passages are giving yields of papillomas that are no greater than those in the earlier passages. It would therefore appear that there has been no further adaptation of the virus in spite of repeated application of the measures that were used when adaptation occurred in the first passage.

The generally low infectivity of the transmissible papillomas and the apparent lack of further adaptation during passage may, however, reflect a fundamental difference in the nature of the infection in cottontail and domestic rabbits. Shope (1935) has suggested that papillomas in the domestic rabbit contain a lower concentration of available virus than in the cottontail rabbit. Moreover, Beard, Bryan and Wyckoff (1939) have isolated by centrifugalization a heavy protein from cottontail papillomas in quantities proportional to infectivity and varying from 0·008 mg. to 1·0 mg. per g., but they have obtained no such protein from papillomas in the domestic rabbit. Bryan and Beard (1949) have also calculated that the number of units of this heavy protein corresponding to a 50 per cent end-point infectious unit is about 94 million. As Friedewald (1942) points out, this figure probably expresses the difficulty of bringing virus in association with susceptible cells, but its magnitude along with the failure to detect virus protein in domestic rabbit papillomas would suggest that the low infectivity of papillomas in the domestic rabbit is due to a low concentration of virus in the papilloma cells. This view has also been supported by serological observations, which have shown that less circulating antibody is produced by the infection in the domestic rabbit than in the cottontail (Kidd, 1938*a*), and that extracts of papillomas from the domestic rabbit contain less specific antigen than those from the cottontail (Kidd, 1938*b*). It is thus possible that the main difference in the nature of the infection in the two species of rabbit is that the lesion in the domestic rabbit is produced with much less multiplication of virus in the affected cells—a phenomenon well exemplified by the bacteriophages, which can produce lysis of bacteria with burst sizes ranging from a very few to 200, representing the amount of multiplication that has taken place within the bacterium (Delbrück, 1942). If this is so it follows that the papilloma virus, once it has gained entry to the susceptible cells, is more virulent to the domestic rabbit, and this would also account for the lesions being of a more progressive nature than in the cottontail rabbit.

Although it would appear likely that there is less multiplication of the papilloma virus in the domestic rabbit, there are undoubtedly other factors that contribute to the difficulty of transmitting these papillomas. Shope (1933) has shown that extracts of domestic rabbit papillomas regularly contain an inhibiting substance which can greatly reduce the infectivity of extracts of cottontail papillomas. It has been suggested that this inhibiting substance may be extravasated antibody, but in the cottontail rabbit this inhibitory effect is observed only in large, disorderly, fissured and inflamed papillomas and when antibody is present in the blood in high titre (Kidd, 1939). Thus, although antibody may sometimes take part in masking the virus, it would appear that some other factor is responsible. It may be that the host-parasite relationship is such that the virus is difficult to extract from the infected cells, or that much of the virus is inactive and proves inhibitory by interfering with the access of active virus to the susceptible cells. In any case it is evident that much work has yet to be done on the questions arising from the apparent paradox of the relative non-infectivity and progressive nature of papillomatosis in the domestic rabbit, and there is no doubt that the transmissible strain described here will provide useful material for the further investigations of these problems.

### SUMMARY.

Shope's finding that infectious papillomatosis of the cottontail rabbit can be transmitted in series in the domestic rabbit has been confirmed.

It has been shown that the transmissible infection behaves in a manner similar to the infection produced by the inoculation of extracts of cottontail papillomas in the domestic rabbit.

The results of the first ten serial passages are described, and the part played by ancillary measures in maintaining the transmissibility of the infection is discussed.

The expenses of this research were defrayed by the British Empire Cancer Campaign.

REFERENCES.

BEARD, J. W., BRYAN, W. R., AND WYCKOFF, R. W. G.—(1939) *J. infect. Dis.*, **65**, 43.
BRYAN, W. R., AND BEARD, J. W.—(1940) *Ibid.*, **66**, 245.
DELBRÜCK, M.—(1942) *Advances in Enzymology*, **2**, 1.
FRIEDEWALD, W. F.—(1942) *J. exp. Med.*, **75**, 197.
KIDD, J. G.—(1938*a*) *Ibid.*, **68**, 703.—(1938*b*) *Ibid.*, **68**, 737.—(1939) *Ibid.*, **70**, 583.
McINTOSH, J., AND SELBIE, F. R.—(1938) *Ann. Rep. Brit. Emp. Campgn.*, **15**, 45.
SELBIE, F. R.—(1946) *Brit. J. exp. Path.*, **27**, 143.
SHOPE, R. E.—(1933) *J. exp. Med.*, **58**, 607.—(1935) *Proc. Soc. exp. Biol., N.Y.*, **32**, 830.
—(1937) *J. exp. Med.*, **65**, 219.